# Evaluating the accuracy of acoustic holograms for precise spatial targeting within the brain

**DOI:** 10.1038/s44384-025-00031-8

**Published:** 2025-10-21

**Authors:** Rachel Burstow, Antonios N. Pouliopoulos

**Affiliations:** School of Biomedical Engineering & Imaging Sciences, King’s College London, London, UK

**Keywords:** Biological physics, Acoustics

## Abstract

Acoustic holography can reconstruct desired pressure fields and overcome phase aberrations caused by refractions in heterogeneous media, such as the skull. However, the accuracy of holographic targeting within the brain has not yet been thoroughly evaluated. We sought to characterize the holographic focusing limits for focused and unfocused single-element transducers. Holographic lenses enlarged the focal size by more than 4-fold. Bifocal lenses achieved foci separation of 7–68 mm and focal depths of 7–83 mm. Lenses were tested in silico and in free-field experiments, with RMS errors of 0.03–0.33. Focused transducers were preferable at low F numbers and were better-suited for murine brain targeting. However, planar transducers can focus over larger areas so have higher clinical relevance in the human brain. Finally, simulations with a human skull showed an RMS error < 0.01. This work provides valuable insight into the accuracy of acoustic holography, demonstrating that transducer design is essential for clinical brain applications.

## Introduction

Holography principles are typically employed to produce optical holograms, where a coherent light source is used to form a 3D visible image^1,2^. However, with the recent advances in computational processing power and additive manufacturing techniques like stereolithography 3D printing, it has become possible to apply these principles in acoustics to create complex pressure fields in a 3D volume^3^. This technology has been used in a range of applications such as 3D ultrasound imaging^4^, contactless power transfer^5^, contactless manufacturing^6^, and many others. Recently there have been investigations into using acoustic holograms for a multitude of biomedical applications^7^ and to induce targeted therapeutic bioeffects, such as blood-brain barrier (BBB) opening^8,9^, or hyperthermia^10,11^.

Ultrasound can propagate through biological tissue without causing damage. It is therefore an invaluable tool in medicine and has been used for a wide range of applications such as ultrasound imaging^12^ and physiotherapy^13^. Focused ultrasound (FUS) therapy is a well-researched tool for minimally invasive therapy^14^. It has the potential to temporarily open the BBB, which can help to treat a wide range of neurodegenerative brain diseases^15–17^. The BBB tightly regulates the transfer of ions, molecules, and cells between the blood and brain parenchyma, protecting the brain from pathogens. However, it can also prevent therapeutic drugs from entering the brain and inhibit lifesaving treatments^18,19^. FUS combined with pre-circulating microbubbles can reversibly, temporarily, and non-invasively open the BBB^20–22^. Acoustic holograms can be used to induce this effect and safely open the BBB, giving them the potential for use in treating conditions such as Alzheimer’s disease^23–25^, Parkinson’s disease^26^, and other neurodegenerative diseases^27,28^. Recently, this was proven possible in symmetric bilateral locations in the mouse^8^ and non-human primate brains^29^. Other technologies such as phased arrays operate in similar ways to produce acoustic holograms, but due to their cost and complexity are not as suitable for widespread use^30^. Acoustic holograms can also more easily be designed to target over larger distances and have a higher spatial resolution since each 3D printed element acts as an individual transducer in the phased arrays.

However, the physical limits of acoustic holograms in the context of preclinical brain applications have not been thoroughly investigated. We aim to demonstrate the versatility of acoustic holograms for brain therapies and identify the limiting parameters during hologram design. We investigated three different types of holograms to test their physical limits. The first investigation tested how large the focal size of a planar transducer could be made. Enlarging the focus size would allow an entire brain region or tumor volume to be treated simultaneously by opening the BBB and delivering drugs to a larger volume. The second investigation involved designing bifocal lenses and increasing the separation between the focus points to demonstrate that different points in the brain can be simultaneously and accurately targeted over pre-clinically and clinically relevant distances in different hemispheres of the rodent or human brain. This could be used for metastatic tumors or if multiple brain regions require simultaneous treatment. This was also tested on a large, focused transducer with a higher frequency to show how the limits vary with different transducer setups. The final investigation involved changing the focal depth of a hologram to demonstrate that different depths in the brain could be targeted without adjusting the transducer position and that large penetration depths can be accurately reached without out-of-target focusing. A comparison between a focused and planar transducer was then conducted by designing holograms for both, whilst keeping parameters such as frequency and aperture the same. This will help guide transducer selection in future applications of acoustic holograms. To date, there has been limited work evaluating the accuracy of acoustic holograms at center frequencies, aperture sizes, dimensions, and types of transducers that are relevant to small-animal and human brain applications. Additionally, the spatial ranges required for large treatment envelopes within the rodent and human brain are far larger than those required for other holographic applications, such as acoustic tweezers or cell patterning^7^.

One of the biggest limitations of FUS in the brain is the scattering effect caused by skull-induced aberrations, which degrade the focusing abilities and the energy delivery within the focal area^31^. Many groups have conducted research into correcting these aberrations^32^. Acoustic holograms can encode medium inhomogeneities into their design using the time reversal method^33,34^. We demonstrated this by designing and testing in silico holograms that are targeted through a human skull. We confirmed that acoustic holograms, when used with the correct transducer and within the limits determined in this study, can improve the focusing ability and field manipulation capabilities and target within a large treatment envelope in the human brain.

## Results

### Focal size acoustic lenses

**Fig. 1:**
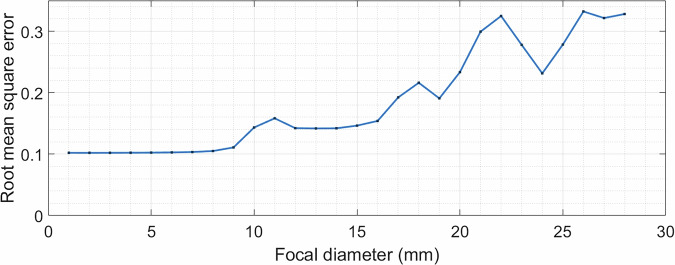
RMSE between the target and simulation 3D pressure maps for a range of focal diameter holograms.

Our first aim was to investigate the ability of a planar transducer to produce sequentially larger focal volumes, so that larger volumes in the brain can be simultaneously treated. A range of lenses with focus diameters between 1 and 28 mm were tested in silico and the root-mean-square errors (RMSEs) between the target and simulation results were plotted in Fig. 1. For this type of hologram, the minimum focus size was 4 mm. This was limited by the frequency of the transducer, which determines the grid size of the simulations. A higher frequency would allow the hologram focal diameter to be smaller. The maximum successful focusing diameter achieved by this transducer aperture was 28 mm, after which there was no clear focusing; a larger diameter transducer would allow for a wider focus hologram to be achieved. However, after 10 mm the energy within the region of interest (ROI) was not evenly distributed, which caused the error to increase.

**Fig. 2:**
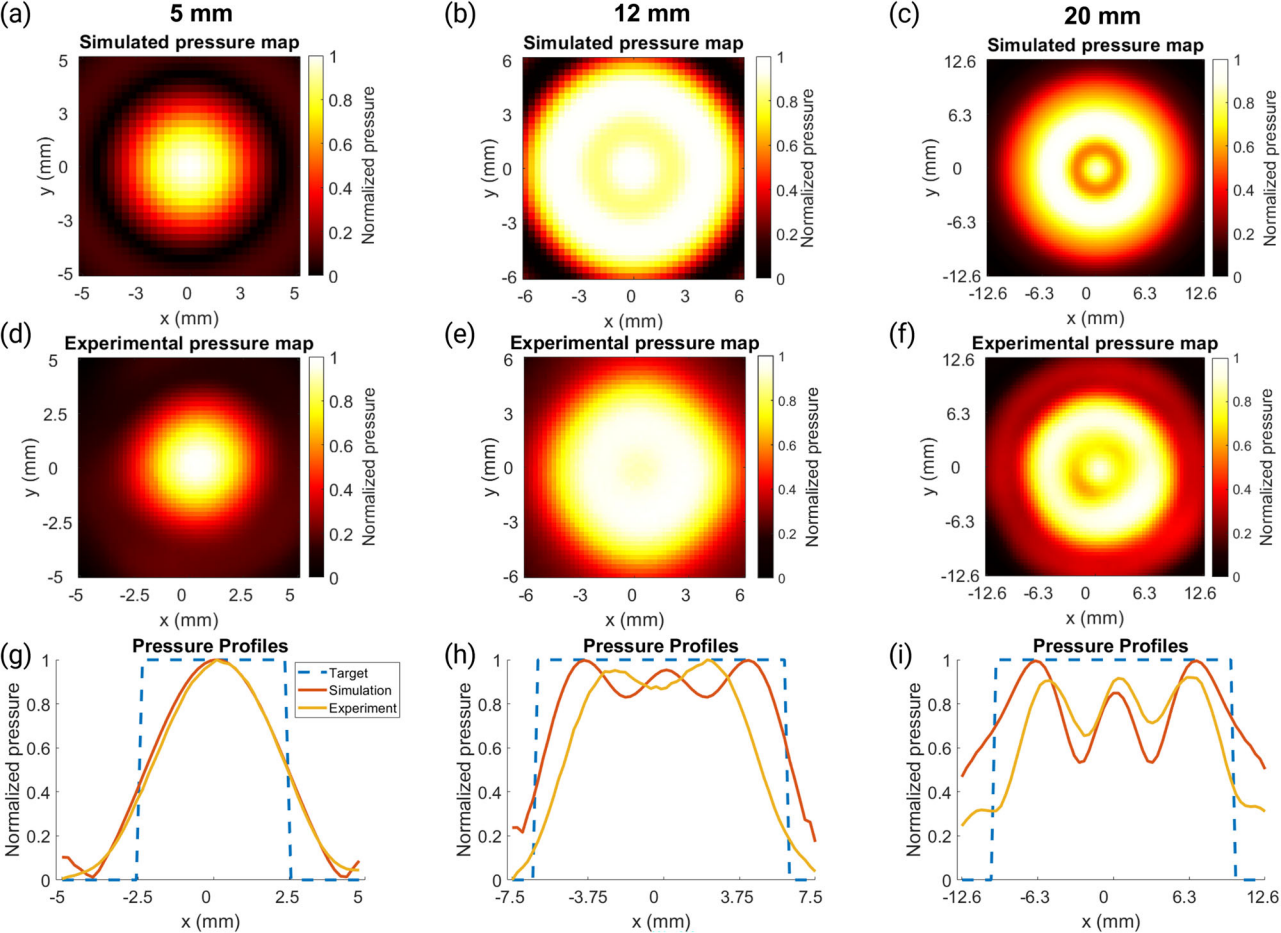
Pressure maps and profiles for holograms with a range of focal sizes. **a**–**c** Normalized peak-negative pressure maps of the simulated holograms for focal sizes of 5 mm, 12 mm, and 20 mm, respectively. **d**–**f** Normalized experimental pressure maps measured with a needle hydrophone for holograms with focal size of 5 mm, 12 mm, and 20 mm, respectively. **g**–**i** Normalized pressure profiles where *y* = 0 mm for the target (blue dashed line), simulation (orange solid line), and experimental results (yellow solid line) for the different focal diameter holograms.

Three holograms were then selected to be manufactured and tested in free field. Figure 2 shows the simulation and experimental results. All three holograms were successful with RMSEs between the 2D simulation and experimental pressure maps of 0.0694, 0.2359, and 0.2037 for the 5 mm, 12 mm, and 20 mm focal diameter holograms, respectively, confirming the robustness and accuracy of the simulation results.

### Bifocal acoustic lenses

**Fig. 3:**
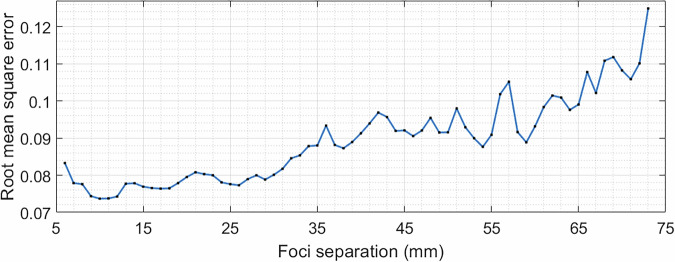
RMSE between the target and simulation 3D pressure maps for a range of bifocal lenses.

The limits of bifocal acoustic lenses were then tested. Figure 3 shows the RMSE values for a range of bifocal lenses from 6 to 68 mm. Below the lower limit of a 7 mm separation, the foci are too close together and start to merge. This is because the resolution of the hologram, which is limited by the frequency of the transducer, is not sufficient to distinguish between them. When the points are too far apart, the aperture of the transducer limits how well the acoustic energy can be spread and as the separation increases, the foci amplitude decreases until there are no longer two distinct points. In our simulations, this happened at 68 mm, which was the upper limit for the bifocal hologram with the 44-mm transducer aperture. A larger aperture transducer would be able to spread the focus points over a greater distance.

**Fig. 4:**
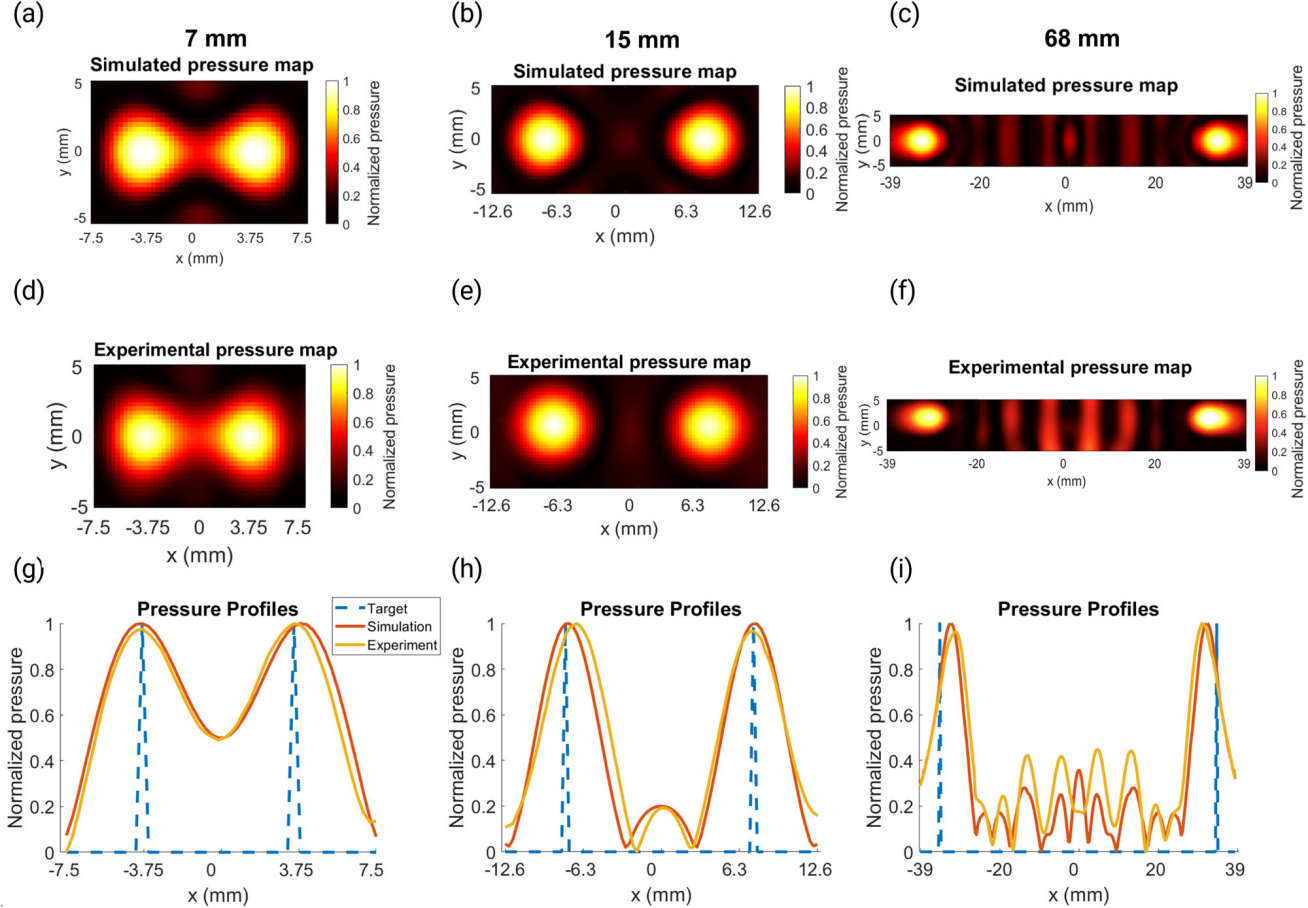
Pressure maps and profiles for the bifocal holograms. **a**–**c** Normalized peak-negative pressure maps of the simulated holograms for foci separations of 7 mm, 15 mm, and 68 mm, respectively. **d**–**f** Normalized experimental pressure maps measured with a needle hydrophone for the corresponding holograms above. **g**–**i** Normalized pressure profiles where *y* = 0 mm for the target, simulation, and experimental results for the 7 mm, 15 mm, and 68 mm foci separations, respectively.

The results of 3 different bifocal lenses are shown below in Fig. 4. The comparison between simulation and experimental results showed very good correlation, with RMSEs of 0.0384, 0.0946, and 0.1198 between the simulation and experimental 2D pressure maps. These holograms show a better correlation and are generally more accurate than the focal enlargement holograms. This is because these holograms do not attempt to increase the focal volume and just relocate the positions of the two foci.

### Focal depth acoustic lenses

**Fig. 5:**
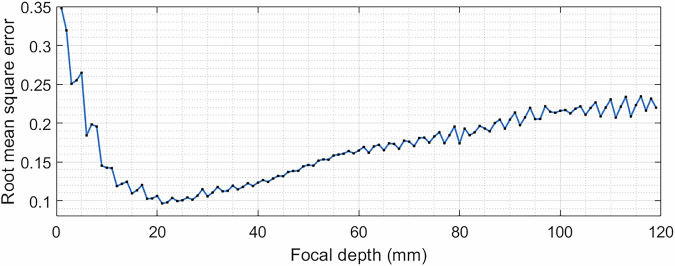
RMSE between the target and simulation 3D pressure maps for a range of focal depths.

Bifocal holograms with a foci separation of 15 mm were designed with a range of focal depths to demonstrate the wide range of penetration depths ultrasound can achieve with this technology. Figure 5 shows the error values between the target and simulation pressure maps over a range of 120 mm focal depths. The lower limit of 15 mm was the closest successful targeting possible. As the depth increased, the focal points slowly became larger and less precise until the upper limit of 83 mm, where a central peak was formed.

**Fig. 6:**
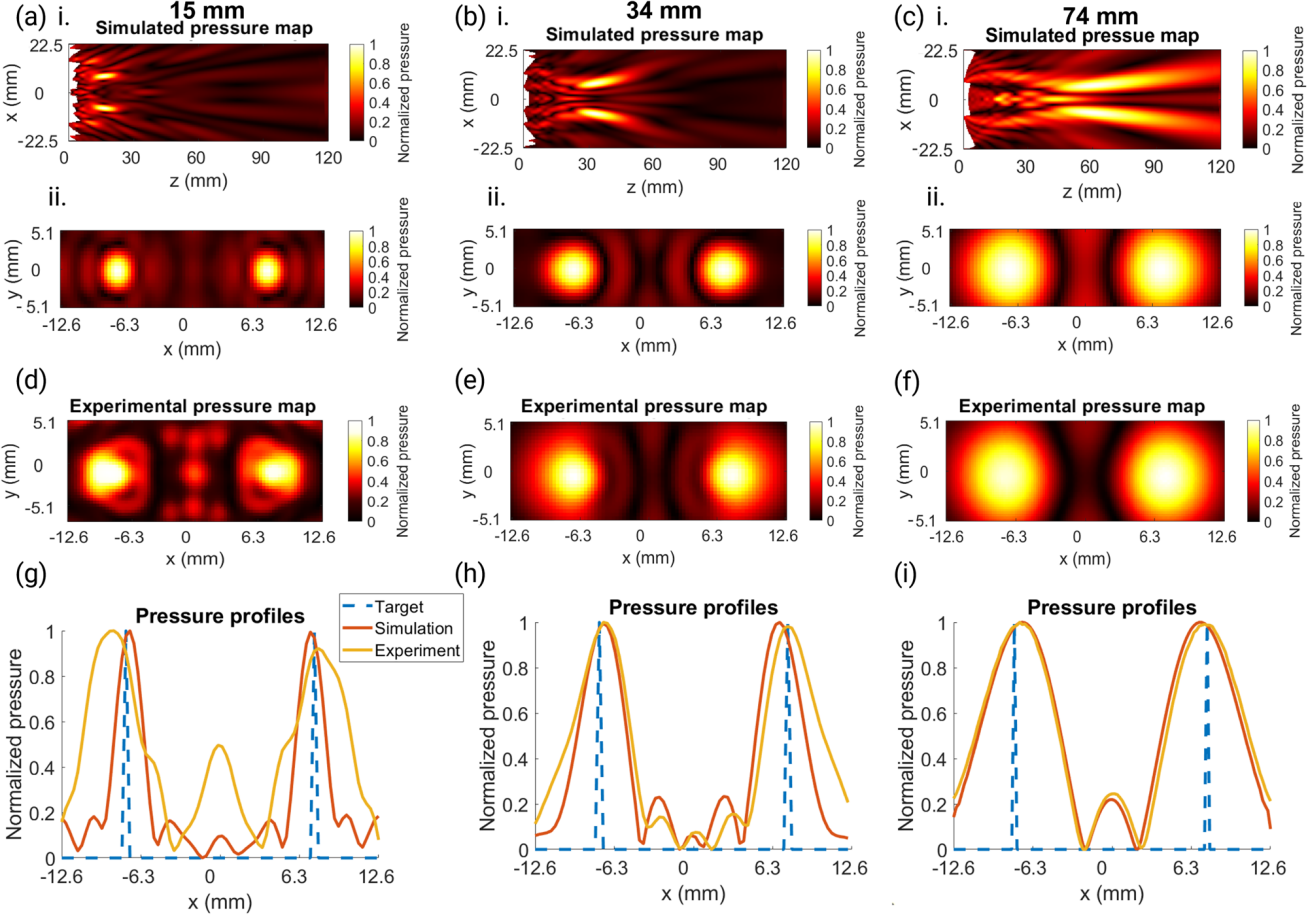
Pressure maps and profiles for varying focal depth holograms. **a**–**c** Normalized peak-negative pressure maps of the simulated holograms for focal depths 15 mm, 34 mm, and 73 mm, respectively. i. XZ-profile and ii. XY-profile. **d**–**f** Normalized experimental pressure maps for the corresponding holograms above. **g**–**i** Normalized pressure profiles where *y* = 0 mm for the target, simulation, and experimental results for the 15 mm, 34 mm, and 74 mm focal depth holograms, respectively.

Focal depths of 15 mm, 34 mm, and 74 mm were then tested experimentally, and the results are shown in Fig. 6. The raster scans were obtained at the respective focal depths, and the holograms successfully created a bifocal pressure distribution at each depth with RMSEs of 0.3330, 0.1649, and 0.0546 between the simulation and experimental 2D pressure maps for the 15 mm, 34 mm, and 74 mm focal depths, respectively. The 15 mm depth hologram was too close to the transducer surface to create circular focal spots, and the waves could not converge fully on the focal points before the image plane, yielding a higher error value. However, there were two clear distinct focal areas, and the deeper penetration depths were more successful.

### Naturally focused transducer

**Fig. 7:**
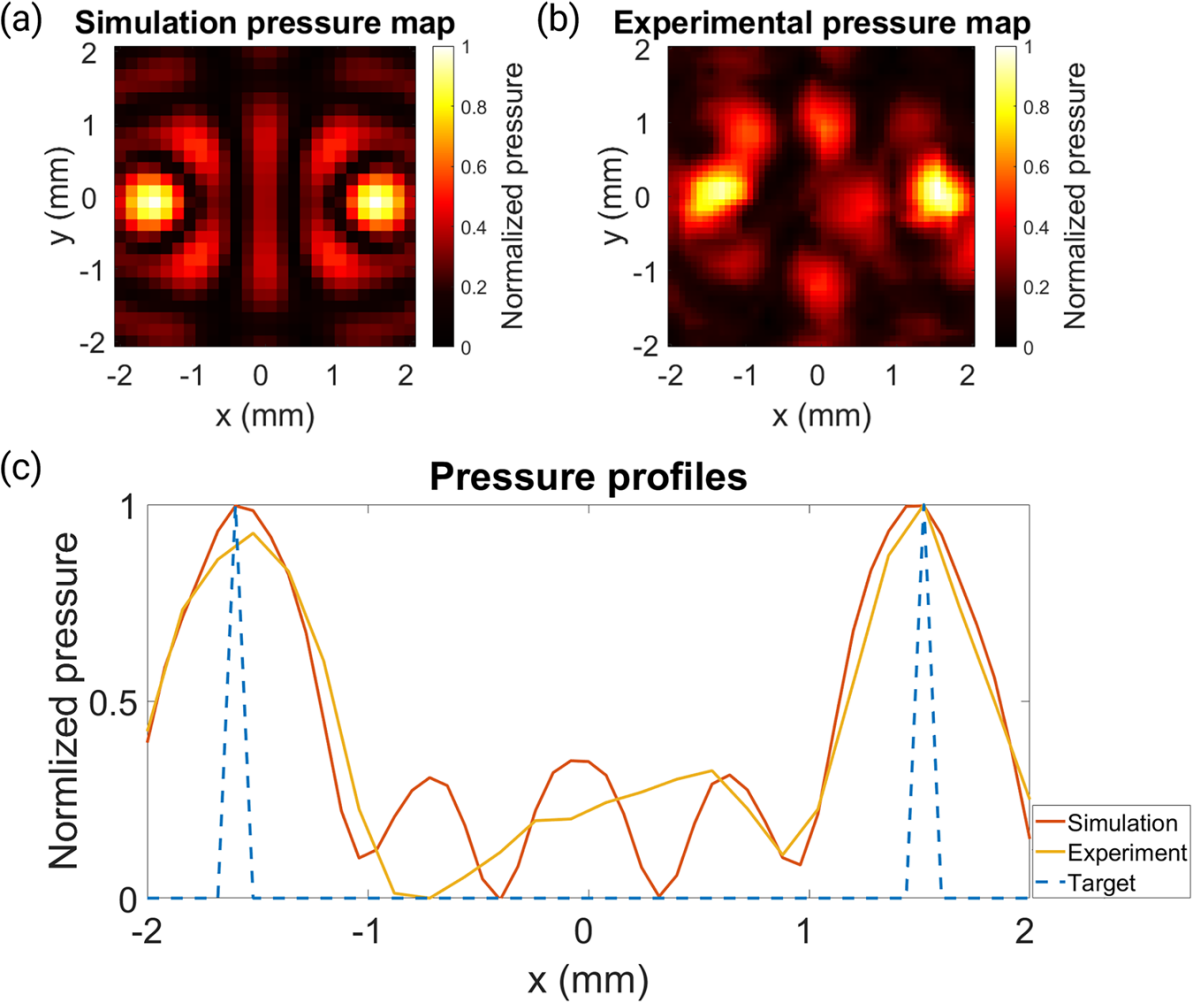
Pressure maps and profiles for a naturally focused transducer. **a** Normalized pressure map for the simulation results of a 3 mm separation bifocal lens. **b** Normalized pressure distribution for the experimental results of the same lens. **c** Comparison of pressure profiles for the simulated, experimental, and target holograms.

The naturally focused transducer used in this study could operate at a higher frequency of 1.645 MHz, which allows for a higher resolution hologram design, due to the shorter wavelength. At this frequency, a bifocal lens with points 3 mm apart (much closer together than can be achieved at 0.5 MHz) at a depth of 50 mm was designed and the results are shown in Fig. 7. This hologram was tested experimentally and an RMSE of 0.1206 was measured between simulation and experimental results. The frequency of the transducer therefore plays an important role in the hologram’s spatial resolution; a higher frequency results in a much finer grid size and a more detailed hologram design.

### Focused vs planar transducer

**Fig. 8:**
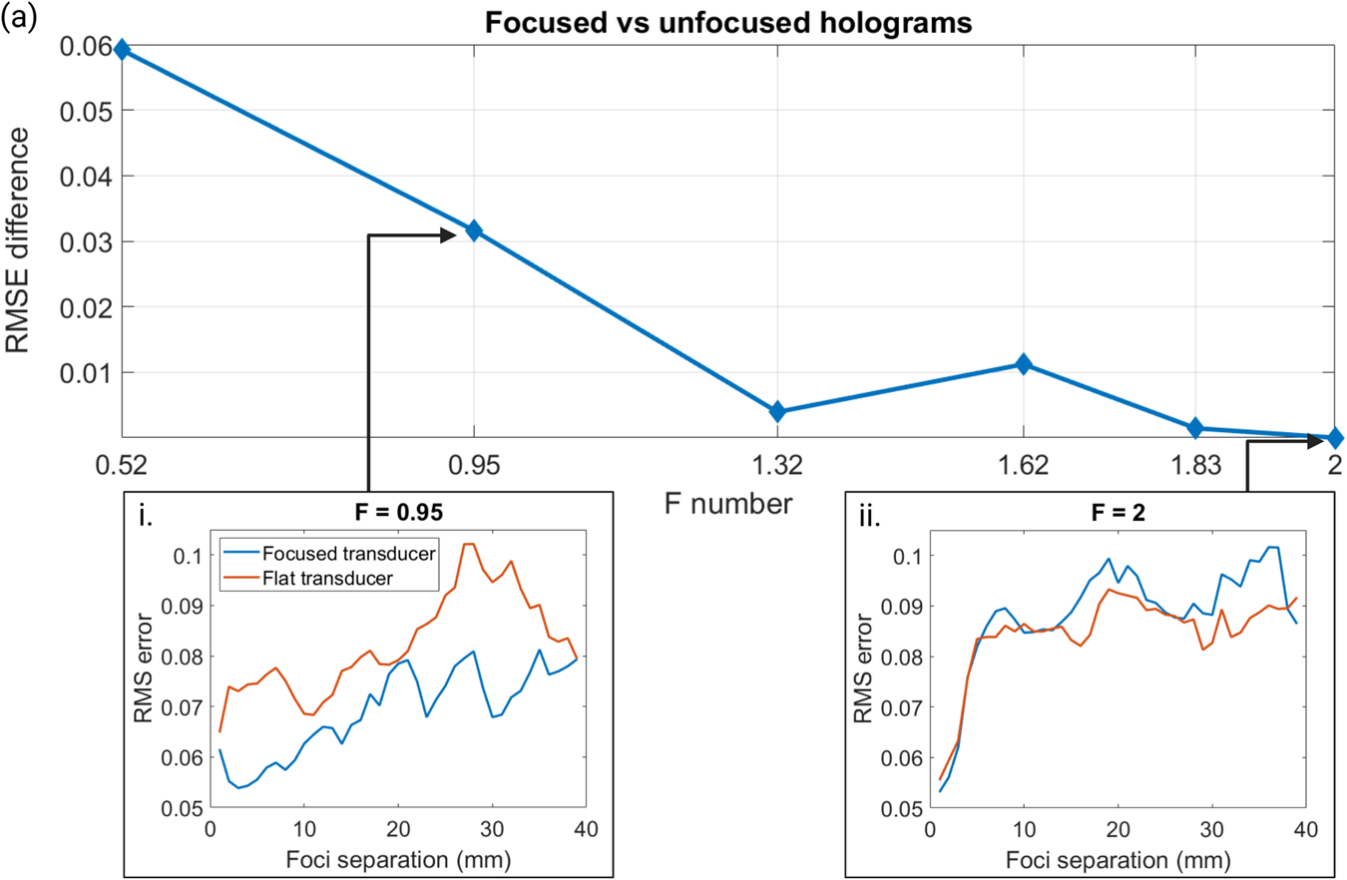
Error values for transducers with a range of F numbers. **a** Difference in error values for a focused and planar transducer with varying F numbers, (i) comparison of RMSE values for the focused and planar transducer bifocal holograms for a range of foci separation at F number 0.95 or focal depth 41.8 mm, (ii) comparison of RMSE values for the focused and planar transducer bifocal holograms for a range of foci separation at F number 2 or focal depth 88 mm.

To generalize the results of this report and inform the transducer selection for hologram design in the future, the impact of the hologram F number was investigated. Holograms were designed for the planar transducer and compared to a range of holograms for naturally focused transducers with the same parameters. Bifocal holographic lenses for curved transducers were designed in silico at the same frequency of 0.5 MHz and aperture of 44 mm, the same as the planar transducer used in the previous sections. Transducers with F numbers ($F=f/d$ where $f$ is the focal depth and $d$ is the aperture) from 0.52 to 2 were used to design bifocal holograms with foci separation of 25 mm, because this was demonstrated as a reliable hologram design for the planar transducer. The RMSEs for these holograms were compared to the RMSE for planar transducer holograms with the same focal depths and the difference in error values is plotted against the F numbers in Fig. 8. This graph demonstrates that for a lower F number, a curved transducer is preferable over the planar transducer, because the narrower beam width allows for a higher lateral resolution and therefore higher accuracy. However, as the F number increases, the difference between the planar and curved transducer reduces and the hologram produces very similar RMS errors. This is further demonstrated by panels (i) and (ii) of Fig. 8, where the error values for a range of foci separations for bifocal holograms are plotted for the focused and planar transducers. For *F* = 0.95, there is a significant and constant decrease in error values when a curved transducer is used instead of the planar transducer over a range of bifocal holograms. However, for *F* = 2, the error values for the focused and planar transducer are very similar and, given the simpler computational design of a holographic lens for a planar transducer, there is no advantage in using a focused transducer for higher F numbers.

### Brain application

It is important to consider the impact of inhomogeneities, such as brain tissue and the unique shape of the patient’s skull, on the ultrasound focusing when designing acoustic holograms. To address this, holograms were designed to test the focusing accuracy within the human skull.

**Fig. 9:**
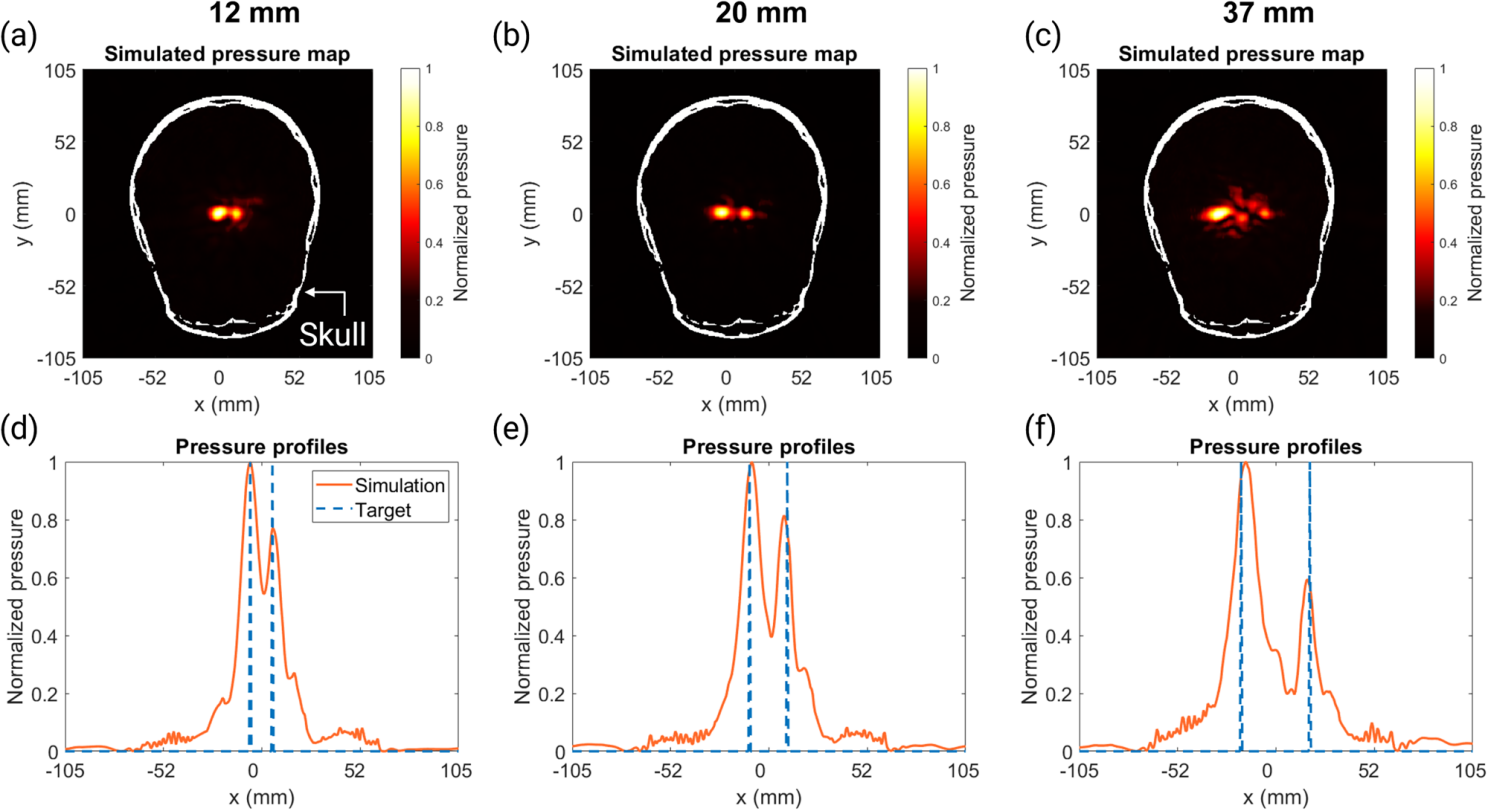
Pressure maps and profiles for holograms targeting through a human skull. **a**–**c** Normalized peak-negative pressure maps of the simulated holograms in the human skull for bifocal lenses with foci separations of 12 mm, 20 mm, and 37 mm, respectively. **d**–**f** Normalized pressure profiles where *y* = 0 mm for the target and simulation results for the 12 mm, 20 mm, and 37 mm bifocal holograms, respectively.

The limits for the bifocal lenses for a flat transducer within the brain were tested in silico. Figure 9 shows the pressure distributions produced by holograms designed for 12 mm (the lower limit), 20 mm, and 37 mm (the upper limit) focal separations. The average RMSEs over the entire head volume were 0.0084, 0.0087, and 0.0089, respectively. There was limited out-of-target focusing within the brain, and the holographic lenses designed have successfully overcome skull aberrations over a range of focal separations. However, there was unevenness in focal amplitudes due to the right-hand side focal spot’s position over a thicker part of the skull. This could be mitigated by adjusting the transducer positioning to achieve more symmetrical focusing. The skull also limits the range of bifocal lenses possible compared to the range used in Section “Bifocal acoustic lenses”. However, by adjusting parameters such as the frequency and aperture of the transducer as discussed previously, the range of distances covered in the brain could be improved.

These preliminary simulations show that the limits evaluated in this study are relevant to clinical applications, due to the hologram’s ability to overcome skull aberrations and spread focusing over two hemispheres in the brain. These pressure distributions were achieved with a single-element transducer and monolithic lens, demonstrating that this technique has huge potential in clinical applications to induce bioeffects over multiple points or large volumes simultaneously.

## Discussion

This study has evaluated and tested the physical limits of acoustic holograms and compared the holograms of curved and planar transducers using therapeutically relevant center frequencies. It was found that, so long as a sufficiently fine grid size and time array was used in the design simulations, the transducer parameters were the biggest contributor to the hologram's versatility. For example, the aperture size limits the width of the hologram design, the frequency limits the resolution of the hologram itself, and a transducer with a larger surface area improves the complexity of the hologram that can be designed.

The curved transducer is better suited to murine in vivo experiments because, unlike the planar transducer, it can accurately focus on points within the small dimensions of the mouse brain. Such transducers can typically incorporate an inner opening, which allows for a passive cavitation detector (PCD) to be used during treatment, to monitor cavitation activity in the brain in real-time. However, the planar transducer is capable of accurately targeting longer distances relevant for the human brain and is computationally faster and simpler to design due to the larger grid size used.

The frequencies used in this study have been proven safe to use in the brain^35–37^, but had not been previously tested for their physical limits in terms of holographic focusing. This study proves that acoustic holograms can target multiple locations over large areas or increase the focal volume of a transducer by more than two-fold, at a range of depths. Acoustic holograms could allow therapeutic ultrasound to be used more precisely to deliver drugs or induce hyperthermia to specific locations in the body, previously unattainable with alternative techniques.

Despite the insightful observations, our study has several limitations. First, experiments were performed in free-field and were limited to 2D spaces. However, the ability of acoustic holograms to correct aberrations is important in improving the accuracy and safety of ultrasound therapies^32,36,37^. Acoustic holography can address this limitation by including the skull and brain geometry and acoustic properties in the simulations, so that any aberrations are corrected by the lens^34^. This has been shown in simulations, but future work will include testing aberration correction in transcranial holographic focusing ex vivo over the range of physical limits. This will pave the way for future pre-clinical and clinical trials where the brain can be targeted to induce localized bio-effects. With acoustic holograms, these treatments could be personalized and become simpler by simultaneously covering large distances within the patient’s brain, and reducing the required number of treatments sessions.

Another limitation of this study is the use of normalized data for the pressure distributions presented. Before in vivo studies or clinical trials can be conducted, the acoustic holograms will need to be calibrated to ensure the required pressure levels or temperatures for treatments can be reached. Additionally, the calibration will need to confirm that no excessive pressures or temperatures are reached that could cause unwanted damage to tissues. However, whilst these are important aspects in terms of safety, evaluating these effects was beyond the scope of this study. Here, we focused on the physical reach of the holograms being tested and have shown that they cover the relevant distances.

Future work will include in silico design and testing of acoustic holograms for targeting specific 3D brain regions and tumor volumes. We will test holographic focusing on mouse brains for targeted BBB opening in vivo, using MRI to confirm successful and accurate targeting. Based on this study’s findings, it is recommended that a higher frequency of at least 1.5 MHz and a curved transducer is used for the murine studies, so that distinct points in the mouse brain can be successfully targeted. However, for studies on larger brains or larger brain regions, a lower frequency or planar transducer could be more effective. We will also evaluate other metrics for assessing the accuracy of transcranial holographic focusing, such as uniformity, focal intensity, side-lobe/interference reduction, on-/off-target effects, and localized heating.

The physical limits and accuracy of acoustic holograms in the context of pre-clinical and clinical brain applications have not been thoroughly investigated to date. Whilst acoustic holography has been used for a range of applications, it has not yet been determined which parameters, such as frequency and transducer aperture, limit them and how much they can manipulate ultrasound fields. This study has shown that holograms, with the correct transducer selection, have the potential to treat a wide range of brain diseases, since they can increase the focal size and can accurately control the focal depth whilst targeting multiple focus points simultaneously over distances required in the human brain. The limits of acoustic holographic lenses were tested in silico and in free-field experiments, which showed very good correlation and proved the targeting ability of the holograms and the robustness of the simulations. A curved transducer with a higher frequency was then tested, which was able to produce a bifocal hologram with points closer together. This is better suited for in vivo studies due to the small size of murine brains. However, the planar transducer provides a simpler design process and can cover relevant distances within the human head. Holograms have the potential to improve the safety and efficacy of brain therapies when used within their physical limits. FUS alone suffers from defocusing and scattering effects caused by the skull. Finally, we used the bifocal lens to demonstrate that acoustic holograms designed using the time reversal method can achieve precise ultrasound manipulation whilst overcoming skull aberrations.

## Methods

### Numerical simulations

The k-Wave MATLAB toolbox was used to run time reversal^38,39^ simulations to design phase-only lenses and test them in silico^40^. A Courant-Friedrichs-Lewy (CFL) number of 0.2 was used to determine the time array. Grids with 7 and 9 points per wavelength were used based on the convergence test for the curved and planar transducer, which resulted in grid spacing of Δh = 0.426 mm and Δh = 0.099 mm, respectively. We simulated two ultrasound transducers operating at 0.5 MHz or 1.645 MHz, which are typical center frequencies used for targeted BBB opening in vivo^41–43^: (i) planar transducer (TX_0.5_44, aperture: 44 mm; Precision Acoustics, Dorcester, UK), and (ii) focused transducer (H-204, aperture: 82 mm, radius of curvature: 63.2 mm; Sonic Concepts, Bothell, WA, USA). Transducer characteristics were given by the manufacturer and were used as inputs for the numerical simulations.

The lens design simulations were based on the techniques used in refs. ^8^^,^^44^. The holographic plane was set to be the exit plane of the transducer, and the height values of each pixel were calculated relative to this plane. For the unfocused transducer, this plane was the transducer emitting surface, so only one simulation was needed to design the holographic lens. However, for the curved transducer the holographic plane was the exit plane of the transducer housing, hence a second simulation was needed to overcome the curvature of the transducer. These two simulation results were combined and then used to calculate the height value of each pixel^8^.

For the increased focus diameter, the target locations were set as a binary disc, the bifocal lenses were designed with 2 monopoles as the target, and the focal depth lenses were designed with a 15 mm bifocal lens, with the target location at varying depths in the *z*-direction. The phase information was recorded at the holographic plane and the conjugate at the working frequency was used. The below equation gives the recorded field information $T(x,y)$ in terms of the height value ($h\left(x,y\right)$) at each pixel in the x-y plane.

$$T(x,y)=\frac{2Z{e}^{-i{k}_{0}\left(d-h\left(x,y\right)\right)}}{2Z\cos \left({k}_{L}h\left(x,y\right)\right)+i\left({Z}^{2}+1\right)\sin \left({k}_{L}h\left(x,y\right)\right)}$$

where $Z=\,{Z}_{L}/{Z}_{0}$ is the normalized impedance (${Z}_{0}={\rho }_{0}{c}_{0}$ is the impedance of water and ${Z}_{L}={\rho }_{L}{c}_{L}$ is the impedance of the lens material). ${k}_{0}=\,\omega /{c}_{0}$ and ${k}_{L}=\,\omega /{c}_{L}$ are the wave numbers of the water and lens material, respectively. $d$ is the distance from the bottom of the lens. A cubic-spline interpolation method was used to select the height values for each pixel’s phase information, thus forming the topography of the lens design. These simulations were run using a GPU cluster (NVIDIA A100-SXM4-40GB).

A final simulation was run to test the lenses in silico. For the purposes of this work, the results were normalized, the average RMSE over the entire grid volume was calculated, and any values over 0.5 of the normalized range were considered to be the focusing of the lens. All values below 0.5 were discarded.

### Free-field experiments

**Fig. 10:**
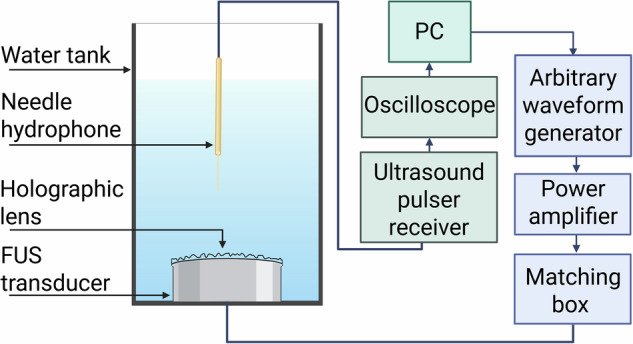
The experimental setup used for free-field testing of the holographic lenses. The lens was attached to the transducer surface and submerged in water. The needle hydrophone was then used to record the pressure distribution of the hologram by obtaining a raster scan along a desired plane.

The experimental setup used for free-field experiments is shown in Fig. 10. The acoustic lenses were 3D printed in ClearResin and a custom-made holder for the transducer and hologram was printed in GreyResin using a Form 3B+ printer (FormLabs, Somerville, MA, USA). The lens and transducer were coupled using ultrasound coupling gel and submerged in deionised and degassed water. An arbitrary waveform generator (33500B waveform generator; Keysight, Berkshire, UK) was connected via a matching box and amplifier to the planar transducer (TX_0.5_44, aperture: 44 mm; Precision Acoustics, Dorchester, UK) or the curved transducer (H-204, aperture: 82 mm, radius of curvature: 63.2 mm; Sonic Concepts, Bothell, WA, USA). The signal was recorded using a 0.5-mm needle hydrophone (NH0500; Precision Acoustics, Dorchester, UK) and processed by a PicoScope (PicoScope 4262; Pico Technology, St. Neots, UK). The hydrophone was positioned at the desired image plane and a 2D raster scan was taken to record the pressure maps produced by the holographic lens. Once the scan had been taken, the 2D pressure maps were normalized and the RMSE between the simulation and experimental maps was calculated by averaging the error between the values at each grid location.

### Skull aberration correction

To model the geometry of the human skull, we used CT data from a female skull at 1 mm resolution (NIH Visible Human Project)^45^ to create a 3D matrix of the skull and brain tissue. We resized the CT image dimensions to fit the simulation grid. This was incorporated within the background grid and used to set the acoustic properties of the skull in the medium of the simulations. The skull CT positioned so that the planar transducer was parallel to the top of the skull. The target locations were then set inside the skull and, during the back propagation simulation, scattering caused by the skull bone was recorded in the phase map, encoding these unique inhomogeneities within the acoustic hologram design.

## Data Availability

Data and code are available on request.
